# Incidence of Takotsubo cardiomyopathy in patients with acute coronary syndrome: a single center retrospective analysis

**DOI:** 10.1186/s43044-024-00542-x

**Published:** 2024-08-26

**Authors:** Tibor Poruban, Martin Studencan, Peter Kirsch, Robert Novotny

**Affiliations:** Eastern Slovak Institute of Cardiovascular Diseases and School of Medicine, Pavol Jozef Safarik University, Kosice, Slovakia

**Keywords:** Acute coronary syndrome, Takotsubo cardiomyopathy, Incidence, Stress

## Abstract

**Background:**

Takotsubo cardiomyopathy (TTC) is an acute and usually reversible condition that often mimics the course of acute coronary syndrome (ACS), making it particularly challenging to differentiate, especially in the initial phases. In this study, we retrospectively analyzed the incidence, clinical course, examination results, and in-hospital mortality of TTC in patients with ACS hospitalized at our cardiology center from January 2018 to October 2023.

**Results:**

During the study period, a total of 3835 selective coronary angiograms were urgently performed at our facility, with a diagnosis of TTC established in 52 (1.35%) patients, the majority of whom were females—48 (93%), with an average age of 64.2 ± 10.2 years. Stress-induced mechanisms were identified in 36 (69%) patients. The most common symptom was chest pain (86.5%). Electrocardiographic changes primarily included ST-segment elevations (61.6%) and depressions (14%). The average left ventricular ejection fraction was 45.1 ± 8.3 (33–57%), typically with an echocardiographic pattern of apical ballooning dysfunction followed by midventricular dyskinesia. In-hospital mortality was zero.

**Conclusions:**

TTC is a reversible condition with a low incidence of complications. Its occurrence in our patient cohort is lower compared to international registries. However, as demonstrated in this study, it is associated with significant in-hospital morbidity.

## Background

Takotsubo cardiomyopathy (TTC) is an entity with clinical manifestations similar to acute coronary syndrome. However, there is no obstruction of coronary arteries, and a characteristic transient dyskinesia of the left ventricular (LV) wall is present in the apical and midventricular segments [1]. LV systolic dysfunction in this case is acute and can be severe, but it is typically reversible with a favorable long-term prognosis. In the acute phase, life-threatening arrhythmias and cardiogenic shock requiring hemodynamic support have been reported in exceptional cases [2]. Emotional or physical stress is considered a key trigger, but the exact etiology of the disease remains unknown. This condition was first described in the Japanese population and later in Europe, North America, and Australia. In this study, we present the demographics, clinical features, and midterm data within our cardiology center.

The aim of this study was to evaluate the incidence of TTC, the rate of left ventricular systolic dysfunction, and in-hospital mortality in patients hospitalized for acute coronary syndrome at the East Slovak Institute of Cardiovascular Diseases in Kosice during a 70-month period.

## Methods

**Fig. 1:**
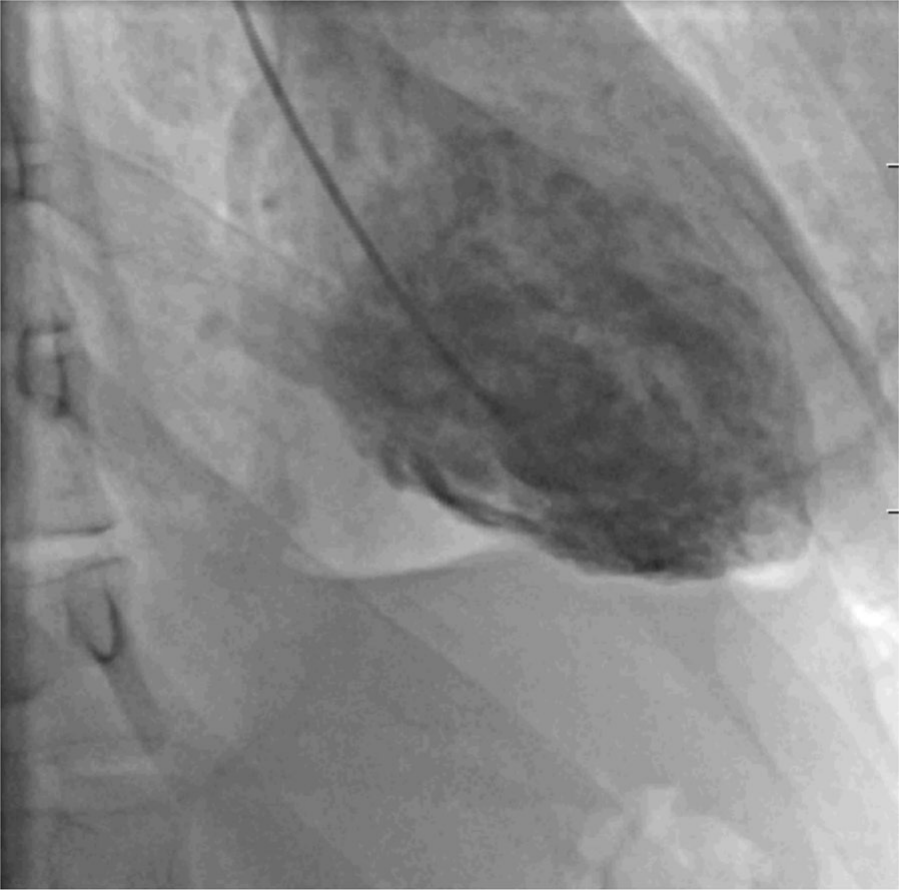
Image of Takotsubo Cardiomyopathy during left ventriculography

The study was descriptive in nature, involving a retrospective analysis of a group of patients who underwent urgent coronary angiography or left ventriculography **(**Fig. 1**)** at the East Slovak Institute of Cardiovascular Diseases in Kosice, from January 2018 to October 2023 (n = 3835). Data were obtained through a retrospective review of patients' medical records. Cases of TTC were considered based on the updated Mayo Clinic criteria: [3]

(I) Transient dyskinesia of the LV walls involving apical and/or midventricular segments, often associated with a stressful trigger,
(II) Absence of obstructive coronary artery disease or angiographic evidence of acute rupture of an atherosclerotic plaque,
(III) New ECG abnormalities such as ST-segment elevation (STE) and/or T-wave inversion or significant elevation in cardiac troponin,
(IV) Absence of pheochromocytoma and myocarditis.

Additionally, patients diagnosed with TTC underwent coronary angiography to exclude significant obstructive coronary artery disease (coronary artery stenosis in the observed dyskinetic LV wall segments > 50%). Demographic data, clinical presentation including triggering events, electrocardiogram, coronary angiographic and echocardiographic findings, cardiovascular biomarker values, management, and concomitant complications were assessed during hospitalization. Within the analyzed dataset, the incidence of TTC, the rate of LV systolic dysfunction, and in-hospital mortality were evaluated from the day of diagnosis. Statistical analysis was performed using SPSS version 27 (SPSS, Chicago, IL, USA). Continuous variables are expressed as median and range, and categorical variables as percentages.

All procedures followed were in accordance with the ethical standards of the responsible committee on human experimentation (Ethics Commitee of VUSCH, Reference Number 17052021) and with the Helsinki Declaration of 1975, as revised in 2008.

## Results

In the specified time frame, a total of 3835 urgent selective coronary angiograms and left ventriculographies were performed at our institution on patients admitted for acute coronary syndrome. The diagnosis of TTC was established in 52 (1.35%) patients, of whom 48 (93%) were females, and the average age of the cohort was 64.2 ± 10.2 (50–82) years (Table 1).

**Table 1:** Incidence of Takotsubo Cardiomyopathy at Our Institution

Analysis period (months)	70
Number of conducted coronary angiograms	3835
of which takotsubo cardiomyopathies	52 (1.35%)

The most common clinical manifestation of TTC was chest pain (n = 45). Two patients experienced sudden cardiac arrest before hospital arrival, and one developed acute pulmonary edema. High-sensitivity cardiac troponin T was elevated in all patients. The most common preliminary diagnosis was acute myocardial infarction with ST-segment elevation (n = 32), followed by acute myocardial infarction without ST-segment elevation (n = 17). TTC was considered the primary diagnosis in only three cases. The most frequent ECG findings on admission were ST-segment elevations (61.6%), followed by depressions (14%). Inverted T waves and sinus tachycardia were also observed (Table 2).

**Table 2:** Characteristics of Patients with Takotsubo Cardiomyopathy

*Clinical presentation*	
Chest pain	45 (86.5%)
Chest pain with shortness of breath	4 (7.8%)
Sudden cardiac arrest	2 (3.8%)
Pulmonary edema	1 (1.9%)
*ECG changes*	
ST segment elevations	32 (61.6%)
ST segment depressions	8 (14%)
Inverted T waves	4 (7%)
Sinus tachycardia	3 (5.8%)
Other	6 (11.6%)
*Initial diagnosis*	
STEMI	32 (61.6%)
NSTE-ACS	17 (32.6%)
Takotsubo cardiomyopathy	3 (5.8%)
Echocardiographic finding	
Apical Ballooning dysfunction	38 (73%)
Midventricular dyskinesia	14 (27%)

NSTE-ACS, non-ST-elevation acute coronary syndrome; STEMI, ST-elevation myocardial infarction

Echocardiographic findings revealed apical ballooning dysfunction of the left ventricle in 38 patients, while the remaining cases exhibited dyskinesia in the midventricular region. The average left ventricular ejection fraction was 45.1 ± 8.3 (33–57)%. A triggering mechanism was identified in 36 (69%) patients, with emotional stress, such as the death of a loved one or an argument, being the most common (Table 3). The average length of hospitalization was 4 days, with no recorded deaths during hospitalization.

**Table 3:** Identified Triggering Mechanisms of Takotsubo Cardiomyopathy

*Emotional stress*	
Unspecified	10
Argument	8
Death of a loved one	4
Family member's illness	3
Work environment	2
*Physical stress*	
Dance	2
Gym	1
Prolonged vomiting	1
Unspecified	5
Unidentified trigger mechanism	16

All patients initially received treatment typical for acute coronary syndrome. In the case of STEMI presentation, dual antiplatelet therapy (acetylsalicylic acid + prasugrel/ticagrelor/clopidogrel) + unfractionated heparin was initiated. If ST elevations were absent, either acetylsalicylic acid monotherapy or in combination with low-molecular-weight heparin was administered. Most patients received beta-blockers and angiotensin-converting enzyme inhibitors during hospitalization.

Perhospitalization course was complicated in five (9.6%) patients by the development of acute pulmonary edema, paroxysmal atrial fibrillation was present in three patients (5.8%), and one patient (1.9%) developed cardiogenic shock requiring hemodynamic support. No deaths occurred in the healthcare facility for any of the patients.

## Discussion

In our study, we analyze a cohort of patients who underwent urgent coronary angiography at our center with suspected acute coronary syndrome from January 2018 to October 2023, ultimately diagnosed with TTC. Stress triggers precede the majority of disease cases. In some instances, life-threatening arrhythmias, cardiogenic shock, or other significant complications may develop, with a favorable prognosis in the medium and long term, along with improved left ventricular systolic function. Recurrence of TTC is rare to exceptional.

The demographic profile of our cohort aligns with the results of previously published studies. Most patients are postmenopausal females. Cases preceded by a stress trigger often involve emotional stress. The precise pathophysiological mechanism leading to TTC has not been described, but elevated levels of circulating catecholamines released during stress appear to be the most probable cause [4]. Other potential causes include microvascular dysfunction, disturbances in fatty acid metabolism, and coronary artery spasm [5]. However, the triggering factor for TTC in the absence of a stress stimulus remains unknown.

Similarly, it remains unexplained why apical segments are more affected in some patients while midventricular segments are more affected in others. One hypothesis involves the disruption of intracellular signaling pathways of ß-1 and 2 types. The activity of ß-2 pathways predominates under physiological conditions, protecting cells from proapoptotic effects of ß-1 adrenoreceptors. In TTC, increased levels of circulating catecholamines likely shift these pathways in favor of ß-1 type, potentiating the activity of evenly distributed adrenoreceptors, thus inducing a negative inotropic effect. Another hypothesis involves reduced fatty acid metabolism in the left ventricular myocardium, as demonstrated by radionuclide imaging [6, 7].

All TTC cases initially presented as acute coronary syndrome, with chest pain being the most common manifestation. Two patients experienced sudden cardiac arrest at the outset, both successfully resuscitated before arriving at our institution. This aligns with the known fact that TTC is associated with repolarization disorders and QT interval prolongation, creating conditions for similar situations, the incidence of which is unknown [8].

The most common ECG abnormality in our patient group was ST-segment elevation (STE). While the opinion that it is a dominant pattern once prevailed, there is now an increasing acknowledgment of its heterogeneity [9]. In other words, not every TTC must initially resemble STEMI. Currently, there is a growing understanding that ECG is not a modality that reliably distinguishes acute coronary syndrome from TTC. This is despite the creation of various electrocardiographic criteria in the past, the challenge being that they were tested only on very small patient groups and are inconsistent across individual studies. These criteria include [10]:

(I) STE in the absence of reciprocal ST depressions
(II) Ratio of STE in leads V4-V6/V1-V3 > 1.25
(III) ST depression in lead aVR with simultaneous absence of STE in lead V1
(IV) STE of at least 1 mm in lead II, with STE in II > III

Complications occurred in 9 (17%) patients, with only one patient developing cardiogenic shock due to severe left ventricular systolic dysfunction, necessitating hemodynamic support. In the context of cardiogenic shock, signs of outflow tract obstruction may be present in some patients, arising from hypokinesis of apical segments and compensatory hyperkinesis of basal segments [11]. It is crucial to consider this when contemplating inotropic or mechanical support, which could exacerbate obstruction. In such cases, beta-blockers may be a better choice, despite the presence of hypotension [12].

In our file, we did not record any cases of death during hospitalization, which is in remote correspondence with the results of international studies where the incidence of in-hospital mortality is lower than in the case of any type of ACS [13]. For completeness—from the data of the Slovak Registry of Acute Coronary Syndromes (SLOVAKS), which deals with the evaluation of diagnostic and therapeutic management of patients with ACS, it follows that in the case of STEMI, we are talking about 4.5%, respectively, in the case of ACS without ST segment elevation 3.6% mortality, with the most common fatal complications being cardiogenic shock and malignant ventricular arrhythmia [14, 15].

Although not observed in our cohort, the presence of wall motion abnormalities (typically apical and/or anterolateral) predisposes to thrombus formation in the left ventricle, potentially leading to systemic embolization. The incidence of left ventricular thrombi is estimated at 5 to 8% based on data from retrospective studies [16]. Cardiogenic shock and embolic complications represent significant TTC-related complications, emphasizing the importance of anticoagulant therapy in this patient group, especially in the acute setting [17].

Electrical abnormalities included ventricular fibrillation in two patients with successful pre-hospital cardiopulmonary resuscitation and atrial fibrillation in three patients with successful amiodarone pharmacological conversion to sinus rhythm.

Echocardiography and cardiac magnetic resonance imaging (MRI) are pivotal imaging modalities in TTC diagnosis and patient health monitoring. The timing of examinations is crucial, ideally performed as soon as suspicion of this diagnosis arises, when there is the greatest chance of detecting regional wall motion abnormalities.

## Limitations of the study

Undoubtedly, the major limitation of our study is its retrospective nature. The assessment of results was partially constrained by the fact that the triggering stressor could not always be unequivocally identified from the documentation. The comparison of our results with other cohorts was limited by the size of our sample. Finally, the lack of the ability to perform timely MRI at our institution would have allowed confirmation or exclusion of the TTC diagnosis in cases that were previously unclear.

## Conclusions

TTC is caused by acute and transient systolic dysfunction of the LV and is typically triggered by a stressor. In this context, it is essential to consider this diagnosis. On the other hand, it is important to emphasize the need for timely coronary angiography in patients with acute chest pain and acute repolarization changes on ECG (especially ST-segment elevations). Although most patients remain hemodynamically stable, complications are not uncommon in TTC patients, and it is crucial to identify and manage them promptly. TTE and MRI play a significant role in the diagnosis, allowing assessment of LV systolic function. Particularly in the case of MRI, its significance lies in enhancing our understanding of the role of microvascular dysfunction in the pathogenesis of this disease. Treatment of TTC primarily relies on angiotensin-converting enzyme inhibitors and beta-blockers. Improving LV systolic function is practically a rule, and the recurrence rate of TTC is exceptionally low. Further studies are needed for a better understanding of the pathogenesis of this nosological entity.

## Data Availability

Not applicable.

## Abbreviations

ACS: Acute Cardiac Syndrome
ECG: Electrocardiogram
LV: Left Ventricle
MR: Magnetic Resonance Imaging
STE: ST-segment Elevation
TTC: Takotsubo cardiomyopathy

## Declarations

### Author summary

Takotsubo cardiomyopathy (TTC) resembles acute coronary syndrome (ACS) but lacks coronary artery obstruction, featuring transient dyskinesia in the left ventricular wall. Acute and severe systolic dysfunction is reversible with a favorable long-term prognosis. Triggered by emotional or physical stress, its exact cause is unknown. This study explores single center experience of the demographics, clinical features, and mid-term outcomes of TTC in patients hospitalized for ACS over a 70-month period in a single cardiac center.
